# WDM-UNet: A Wavelet-Deformable Gated Fusion Network for Multi-Scale Retinal Vessel Segmentation

**DOI:** 10.3390/s25154840

**Published:** 2025-08-06

**Authors:** Xinlong Li, Hang Zhou

**Affiliations:** 1 Weihai Campus, Beijing Jiaotong University, Weihai 264402, China; 22721059@bjtu.edu.cn; 2 School of Electronics and Information Engineering, Beijing Jiaotong University, Beijing 100082, China

**Keywords:** retinal vessel segmentation, squeeze and excitation, multi-scale feature interaction, fundus images

## Abstract

Retinal vessel segmentation in fundus images is critical for diagnosing microvascular and ophthalmologic diseases. However, the task remains challenging due to significant vessel width variation and low vessel-to-background contrast. To address these limitations, we propose WDM-UNet, a novel spatial-wavelet dual-domain fusion architecture that integrates spatial and wavelet-domain representations to simultaneously enhance the local detail and global context. The encoder combines a Deformable Convolution Encoder (DCE), which adaptively models complex vascular structures through dynamic receptive fields, and a Wavelet Convolution Encoder (WCE), which captures the semantic and structural contexts through low-frequency components and hierarchical wavelet convolution. These features are further refined by a Gated Fusion Transformer (GFT), which employs gated attention to enhance multi-scale feature integration. In the decoder, depthwise separable convolutions are used to reduce the computational overhead without compromising the representational capacity. To preserve fine structural details and facilitate contextual information flow across layers, the model incorporates skip connections with a hierarchical fusion strategy, enabling the effective integration of shallow and deep features. We evaluated WDM-UNet in three public datasets: DRIVE, STARE, and CHASE_DB1. The quantitative evaluations demonstrate that WDM-UNet consistently outperforms state-of-the-art methods, achieving 96.92% accuracy, 83.61% sensitivity, and an 82.87% F1-score in the DRIVE dataset, with superior performance across all the benchmark datasets in both segmentation accuracy and robustness, particularly in complex vascular scenarios.

## 1. Introduction

The retinal vasculature serves as a critical diagnostic window for both ocular and systemic pathologies, as its morphological alterations provide clinically actionable biomarkers. For example, glaucoma [1,2] is associated with central retinal vessel displacement and peripheral vascular attenuation. Diabetic retinopathy [3] is characterized by abnormal neovascularization, microaneurysms, and hemorrhages, whereas age-related macular degeneration (AMD) [2] presents with macular vascular anomalies and deposit formation. The morphological features of the retinal vasculature serve as essential diagnostic criteria, enabling the early detection and precise characterization of vascular lesions. These assessments are critical for informing disease prognoses and guiding therapeutic interventions. The accurate analysis of these vascular features enhances clinical evaluation and early diagnosis, highlighting the need for robust retinal vessel segmentation algorithms in computer-aided diagnostic systems.

Fundus vascular segmentation in the spatial domain has been a central focus of recent research. Early studies primarily utilized traditional image-processing techniques, such as thresholding (both global and local) [4], Canny edge detection [5], and region-growing methods [6]. However, these approaches exhibit limited robustness, as they are highly sensitive to factors such as illumination variations, shadows, and noise. Moreover, they are inherently unable to capture high-dimensional semantic information, rendering them ineffective at addressing vessel overlaps and occlusions commonly observed in fundus images. Manual parametrization in algorithm design restricts adaptability across varied clinical scenarios, resulting in substantial diagnostic variability and increasing the risk of erroneous edge detection, which may lead to misdiagnosis. Meanwhile, the advent of convolutional neural networks (CNNs) has transformed fundus imaging, with U-Net architectures significantly enhancing segmentation accuracy.

The Visual Transformer (ViT) [7] addresses the local constraints of convolutions by partitioning images into sequential blocks, thereby enabling global feature modeling. The optimal integration of U-Net and ViT architectures can reduce the model’s complexity while improving the global contextual capture. For instance, Li et al. [8] developed a Hybrid Dense U-Net (H-DenseU-Net) that combines 2D spatial features with 3D contextual information. However, challenges persist in modeling complex spatial hierarchies across diverse imaging conditions. Similarly, Qu et al. [9] introduced a dual-path network with multi-scale feature aggregation, enhancing both main vessel detection and peripheral feature extraction. Nevertheless, these convolution-based spatial domain approaches remain fundamentally limited by (1) restricted receptive fields, which hinder the segmentation of context-dependent structures; (2) poor scale adaptation, affecting the concurrent detection of capillaries and major vessels; and (3) noise sensitivity, which impairs textural discrimination in low-quality fundus images.

To overcome the limitations of local feature extraction in the spatial domain, frequency-domain segmentation offers an alternative approach that effectively captures multi-scale global features often missed by conventional convolutions. Frequency-domain analysis decomposes images into distinct spectral components through transformation techniques, such as Fourier and wavelet transforms. This decomposition enables comprehensive signal analysis across multiple scales: Low-frequency components (LL) preserve structural information, while high-frequency components (HL, LH, and HH) retain fine details, including edges and textures [10].

GFNet [11] employs a two-dimensional discrete Fourier transform (2D DFT) to map features from the spatial to the frequency domains and conducts representation learning through filtering. However, this method extracts frequency information along a single axis, resulting in incomplete global information capture and neglecting the critical role of local information in image feature extraction. MEW-Unet, proposed by Ruan et al. [12], performs Fourier transformation across three axes of input features and allocates external weights in the frequency domain through a weight generator. Despite its innovative approach, this method relies primarily on basic filtering properties for frequency-domain analysis. WRANet, introduced by Zhao et al. [13], integrates wavelet transforms with residual attention, decomposing CNN features into low- and high-frequency components using a discrete wavelet transform. However, indiscriminately eliminating high-frequency components may compromise critical feature information, potentially causing the loss of retinal vascular details. In fundus image segmentation, high-frequency components capture fine structures, such as microvessels, while low-frequency components provide the overall retinal morphology. Therefore, to enhance the model’s sensitivity to pathological changes at different scales, it is essential to improve the local detail extraction while preserving the global structural integrity.

Compared to purely spatial domain methods, frequency-domain analysis more effectively distinguishes the key structures from noise, thereby enhancing the model’s noise robustness. Low-pass filtering suppresses high-frequency noise while retaining the retinal morphology, enabling the stable segmentation of lesion areas in complex backgrounds. Wavelet convolution, combining time- and wavelet-domain analyses, expands the receptive field of convolutional neural networks (CNNs) and achieves more precise extraction of both high- and low-frequency information. Unlike traditional convolution, this method employs a cascading approach to enhance CNNs’ responsiveness to low-frequency features.

To overcome these challenges, we propose WDM-UNet, a frequency-domain scale fusion model that simultaneously optimizes the local detail extraction and global structure modeling by integrating spatial and frequency-domain features. The encoder stage comprises a Deformable Convolution Encoder (DCE) and a Wavelet Convolution Encoder (WCE), each responsible for distinct feature representations. The DCE enhances local feature modeling through dynamic receptive fields, improving the detection accuracy for complex vascular structures, such as crossing and curved vessels, while preserving fine-grained details, including vessel trajectories and irregular boundaries. This design ensures robust adaptation to heterogeneous vascular networks.

The WCE captures global structural and semantic information through low-frequency CNN features while preserving local details across layers through residual connections. Hierarchical discrete wavelet convolution further enhances deep feature extraction. The model maintains a U-shaped architecture, where the DCE and WCE perform shallow-layer downsampling with direct feature fusion. For deeper feature integration, the Gated Fusion Transformer (GFT) module employs attention-based gated fusion operations to optimize multi-scale feature aggregation.

In the decoder stage, depthwise separable convolution is used, with multi-scale feature propagation achieved through skip connections to mitigate edge detail loss during pooling operations. The initial image characteristics are preserved through a hierarchical fusion strategy that directly propagates shallow-layer features to corresponding decoder upsampling stages. This design optimizes the computational efficiency while facilitating long-range dependency learning.

We evaluated this model in three commonly used public datasets of fundus images, demonstrating substantial improvements over existing methods.

Our main contributions are summarized below:

- A novel retinal image segmentation architecture known as WDM-UNet is proposed, which integrates feature extraction and fusion from both spatial and wavelet domains, emphasizing local details while preserving global contextual information;
- The wavelet transform is incorporated to enhance the receptive field, enabling multi-scale information analysis and frequency-domain image processing. This approach leverages low-frequency components for global feature extraction and high-frequency components for fine-detail restoration;
- A GFT mechanism is designed to merge features from dual encoders via an attention mechanism, balancing the intricate details with the structural integrity and maintaining equilibrium between global and local feature representations;
- Separable convolutions are implemented in the decoder stage, and skip connections utilizing features from different scales in the shallow network layers are employed, thereby capturing various vessel dimensions while simultaneously reducing the computational complexity and preserving the local feature integrity.

## 2. Method

A novel Wavelet-Deformable Mixture U-Net (WDM-UNet) architecture is proposed for medical image segmentation, integrating spatial and wavelet domain representations via four optimized components: (1) a Deformable Convolution Encoder for local feature adaptation, (2) a Wavelet Convolution Encoder for spectral decomposition, (3) a Gated Fusion Transformer with attention mechanisms, and (4) a depthwise separable convolution decoder. This architecture achieves simultaneous global contextual modeling and local detail preservation through hierarchical multi-scale fusion, offering particular advantages in boundary reconstruction accuracy.

**Figure 1:**
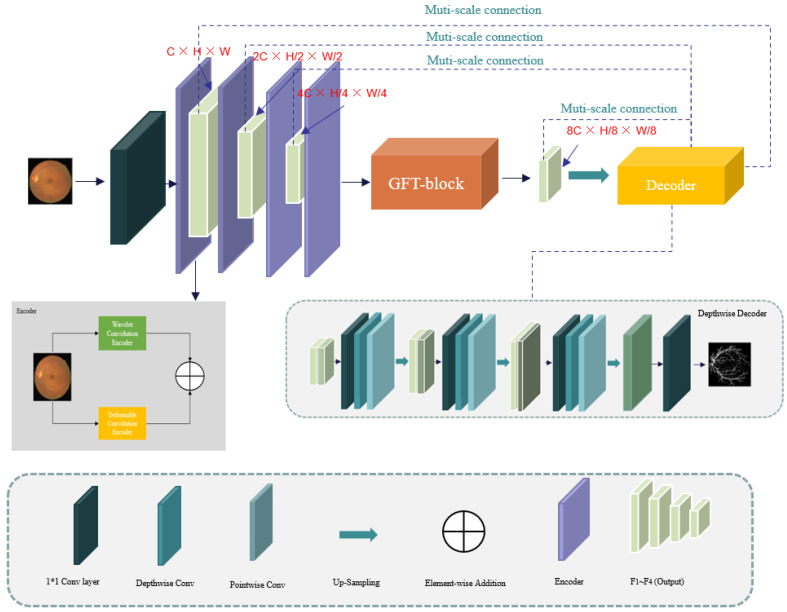
Schematic illustration of the WDM-UNet’s hybrid architecture.

The WDM-UNet architecture, depicted in Figure 1, uses a hierarchical encoder–decoder structure that extends the conventional U-Net framework. This architecture incorporates wavelet convolution for frequency-domain analysis in the encoding stage, enhancing feature extraction from low-frequency components to optimize the global contour delineation. Skip connections between corresponding encoder and decoder layers enable multi-scale analysis through depthwise separable convolution operations.

The proposed architecture features a three-part encoder structure with six hierarchical units. Spatial information from deformable convolution and frequency-domain features from wavelet convolution are fused in the first two layers. The deepest layer uses a gated fusion transformer (GFT) with self-attention mechanisms to optimize the feature utilization efficiency. These skip connections connect the encoder and decoder stages, enabling multi-scale feature integration and the recovery of the spatial information lost during downsampling. The symmetrical decoder consists of three hierarchical layers, each integrating corresponding encoder outputs through upsampling operations that double the channel’s dimensionality. The following sections provide detailed descriptions of each architectural component.

### 2.1. Deformable Wavelet Encoder Module

#### 2.1.1. Deformable Convolution Encoder

The Deformable Convolution Encoder (DCE) component maintains the U-Net encoder’s hierarchical structure while introducing critical modifications to enhance feature extraction. Using successive convolution layers with progressively increasing receptive fields, the DCE captures multi-scale representations while maintaining the computational efficiency. This key innovation replaces conventional convolution operations with deformable convolutions that learn spatial sampling offsets. This adaptive sampling enables the precise localization of the retinal vasculature by dynamically adjusting to vessel morphology variations and pathological deformations.

By dynamically adjusting the receptive field, deformable convolution better accommodates vascular deformations and complex local structures, particularly at vessel bifurcations, tortuous segments, and pathological regions. In contrast, conventional convolutional neural networks (CNNs) rely on fixed geometric receptive fields, limiting their ability to model the elongated, curved, and deformable nature of the retinal vasculature. The DCE addresses this constraint through learnable spatial offsets, enabling adaptive feature extraction.

**Figure 2:**
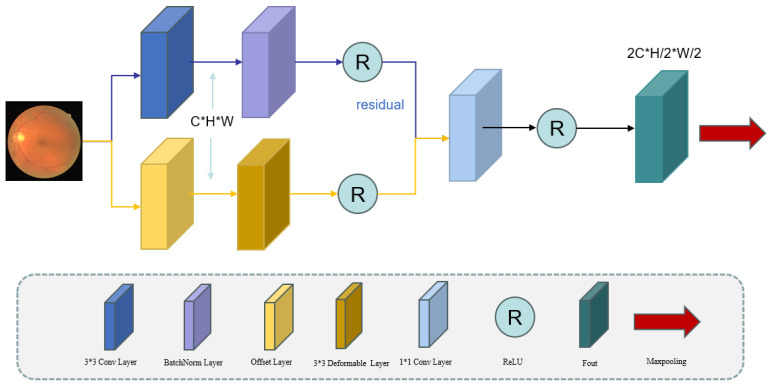
Schematic of the deformable convolution encoder’s architecture.

As shown Figure 2, the DCE uses parallel processing streams: a primary branch, using standard 3 × 3 convolutions with batch normalization (BN) and activation functions for stable gradient propagation and global feature extraction, and a secondary branch, using offset prediction layers combined with deformable convolutions for local feature enhancement. These components are connected through residual connections, effectively combining global contextual information with local deformation features to improve the segmentation accuracy. The channel’s dimensionality is standardized using 1 × 1 convolutions [14,15,16]. The encoder uses max pooling for downsampling, with two pooling operations across three layers to progressively expand the receptive field while maintaining the computational efficiency. The key innovation is the parallel deployment of deformable convolutions, which enable dynamic learned sampling through spatial offsets, complementing conventional convolution operations. In the residual pathway, the input first undergoes a conventional 3 × 3 convolution operation, transforming the channel’s dimensionality from C to C’ during the feature extraction.

(1) $$y_{\mathrm{conv}}\left(p\right)=\sum_{p_{n}\in R}w_{\mathrm{conv}}\left(p_{n}\right)\cdot x\left(p+p_{n}\right)$$

In the deformable convolutional network’s main branch, we generate offsets via the offset computational network as follows:

(2) $$\Delta p=W_{\mathrm{offset}}*x$$

where $W_{\mathrm{offset}}$ is a 3 × 3 convolutional kernel. After the offset convolution computation, the number of image channels increases from C to C′. The deformable convolutional network (DCN) then learns the offsets ($\Delta p_{n})$ to dynamic sampling $(y_{\mathrm{dconv}}\left(p\right)=\sum_{p_{n}\in R}w_{\mathrm{dconv}}\left(p_{n}\right)\cdot x\left(p+p_{n}+\Delta p_{n})\right)$. We then use bilinear interpolation $\left(x(p+p_{n}+\Delta p_{n})\right)$ to enhance the model’s stability and robustness, preventing pixel loss.

The outputs from both processing branches are element-wise summed and propagated through a residual connection, enhancing the gradient flow while mitigating vanishing/exploding gradient issues. This operation can be formally expressed as follows:

(3) $$y_{\mathrm{residual}}=y_{\mathrm{ReLU}}+y_{\mathrm{dconv}}.$$

Finally, a 1×1 convolutional kernel is used to adjust the number of channels. The first layer’s output is obtained by applying the ReLU activation function.

(4) $$\begin{array}{cc} y_{1\times 1} & =W_{1\times 1}*y_{\mathrm{residual}}\\ y_{\mathrm{output}} & =\max(0,y_{1\times 1}) \end{array}$$

Max pooling is applied to $y_{\mathrm{output}}$ for downsampling, increasing the receptive field and capturing more global variables. This process reduces the height and width of the feature maps to H/2 and W/2, respectively. Concurrently, the number of channels (*C*) is doubled to 2C at each layer to capture finer details. This encoder design enables our model to effectively capture target-scale variations and detailed features in the spatial domain.

#### 2.1.2. Wavelet Convolution Encoder

Wavelet convolution combines traditional convolution operations with wavelet transform techniques to capture multi-scale and multi-frequency features through wavelet decomposition. For retinal vessel image-processing tasks, such as vessel extraction and edge detection, wavelet convolution effectively extracts features across multiple scales. Additionally, using deformable convolutional kernels, it adaptively adjusts the receptive field to accommodate local image variations. In this implementation, we employ the Haar wavelet as the mother wavelet function due to its computational efficiency and perfect reconstruction properties for medical image analysis. The Haar wavelet is particularly suitable for retinal vessel segmentation, as it provides effective edge detection capabilities while maintaining a low level of computational complexity. The choice of the Haar wavelet is motivated by its orthogonal property and ability to capture both smooth regions (low frequency) and edge information (high frequency) simultaneously.

**Figure 3:**
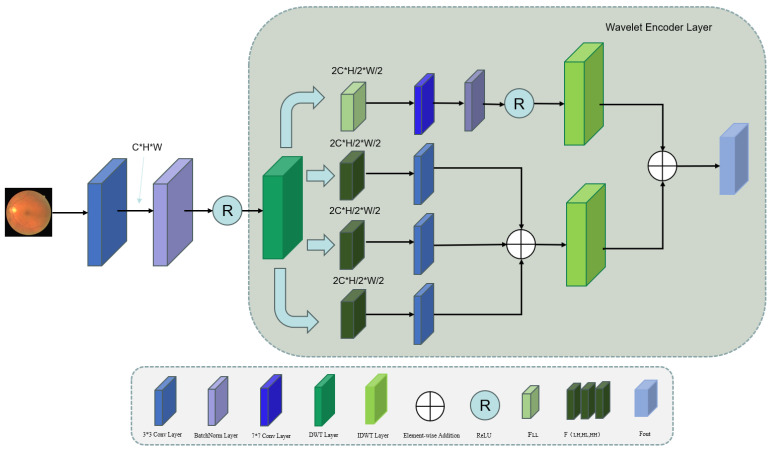
Schematic of the wavelet convolution encoder’s architecture.

As illustrated in Figure 3. Our model uses the Discrete Wavelet Transform (DWT) for multi-scale frequency decomposition, generating distinct spectral components. The approximation coefficients (LL) preserve the retinal image’s structural information, while detail coefficients (LH, HL, and HH) encode vascular branching patterns and localized features [17,18]. We chose the DWT for its combined time–frequency localization properties, computational efficiency, and perfect reconstruction capability. This transform allows simultaneous access to localized high-frequency details and low-frequency structural information, making it particularly suitable for analyzing non-stationary medical images with rich textural details. Moreover, the DWT offers advantages in reducing the computational complexity, enabling effective data compression and providing lossless signal reconstruction. Feature extraction starts with a 3×3 convolution operation, batch normalization, and ReLU activation applied to the input feature map, $F=\sigma (\mathrm{BN}\left(W_{c}*X+b_{c}\right))$. Here, $W_{c}$ represents the 3 × 3 convolutional kernel, ∗ denotes the convolution operation, and $b_{c}$ is the bias term. The function σ(·) represents the ReLU activation function. This layer adjusts the number of channels from *C* to $C^{\prime }$. After this initial processing, the retinal feature image is decomposed into high- and low-frequency components. We then apply the discrete wavelet convolution to obtain four subbands as follows:

(5) $$F_{LL},F_{LH},F_{HL},F_{HH}=\mathrm{DWT}\left(F\right)$$

The DWT is computed as follows:

(6) $$\begin{array}{c} F_{LL}=\frac{1}{4}\left(F_{00}+F_{01}+F_{10}+F_{11}\right)\\ F_{LH}=\frac{1}{4}\left(F_{00}-F_{01}+F_{10}-F_{11}\right)\\ F_{HL}=\frac{1}{4}\left(F_{00}+F_{01}-F_{10}-F_{11}\right)\\ F_{HH}=\frac{1}{4}\left(F_{00}-F_{01}-F_{10}+F_{11}\right) \end{array}$$

The variables $F_{00}$, $F_{01}$, $F_{10}$, and $F_{11}$ correspond to the 2×2 local pixel blocks within the input feature map. The Discrete Wavelet Transform (DWT) doubles the channel count while halving the spatial resolution, producing feature maps of dimensions H/2 and W/2. This transformation includes downsampling. The low-frequency components then undergo standard convolution, batch normalization (BN), and ReLU activation, formulated as $F_{LL}^{\prime }=\sigma \left(\mathrm{BN}\left(W_{LL}*F_{LL}+b_{LL}\right)\right)$.

For the high-frequency components, instead of using a direct residual connection, we retain them for subsequent integration with the processed low-frequency component through the Inverse Discrete Wavelet Transform (IDWT). The IDWT reconstructs the original spatial resolution from the wavelet subbands, preserving both the structural integrity and high-frequency details.

(7) $$F_{\mathrm{wavelet}}=\mathrm{IDWT}\left(F_{LL}^{\prime },F_{LH},F_{HL},F_{HH}\right)$$

The IDWT operation can be represented as follows:

(8) $$\begin{array}{c} F_{\mathrm{out}}\left(2i,2j\right)=F_{LL}^{\prime }\left(i,j\right)+F_{LH}\left(i,j\right)+F_{HL}\left(i,j\right)+F_{HH}\left(i,j\right)\\ F_{\mathrm{out}}\left(2i,2j+1\right)=F_{LL}^{\prime }\left(i,j\right)-F_{LH}\left(i,j\right)+F_{HL}\left(i,j\right)-F_{HH}\left(i,j\right)\\ F_{\mathrm{out}}\left(2i+1,2j\right)=F_{LL}^{\prime }\left(i,j\right)+F_{LH}\left(i,j\right)-F_{HL}\left(i,j\right)-F_{HH}\left(i,j\right)\\ F_{\mathrm{out}}\left(2i+1,2j+1\right)=F_{LL}^{\prime }\left(i,j\right)-F_{LH}\left(i,j\right)-F_{HL}\left(i,j\right)+F_{HH}\left(i,j\right) \end{array}$$

The inverse transform returns the feature dimensions to their original size ($C^{\prime }\times H\times W$), preserving the channel’s dimensionality determined by the initial convolution layer. This reconstruction combines high-frequency components with processed low-frequency features, ensuring that crucial anatomical details, including vascular boundaries and fine-tissue structures, are preserved.

After the inverse discrete wavelet transform (IDWT), the reconstructed feature map undergoes additional convolutional processing for hierarchical feature representation at deeper network levels.

(9) $$F_{\mathrm{final}}=\sigma \left(F_{\mathrm{wavelet}}\right)$$

As the feature map moves through the encoder, its channel’s dimensionality progressively expands, while its spatial resolution systematically contracts, following standard encoder design principles. The dimensional transformations follow a consistent pattern: from the initial $C^{\prime }\times H\times W$ to $2C^{\prime }\times H/2\times W/2$ in the second layer, ultimately reaching $4C^{\prime }\times H/8\times W/8$ in the third encoding stage.

The Wavelet-Enhanced Convolution Encoder (WCE) generates a high-fidelity feature map with enhanced global representations and precisely delineated contours. It provides superior noise suppression, effectively reducing irrelevant artifacts while preserving diagnostically relevant features. By combining wavelet decomposition and multi-scale reconstruction, the model extracts hierarchical feature representations, which are particularly advantageous for retinal imaging, where illumination heterogeneity and noise contamination are prevalent. This wavelet-optimized framework significantly reduces noise-induced segmentation inaccuracies while maintaining the topological consistency of the vascular network. As a result, the WCE achieves state-of-the-art performance in retinal vessel segmentation and diabetic retinopathy (DR) diagnosis, enabling the precise localization of microaneurysms and pathological lesions.

### 2.2. Gate Fusion Transformer Block

The feature representations generated by the Deformable Convolution Encoder (DCE) and Wavelet-Enhanced Convolution Encoder (WCE) capture high-level semantic features of the retinal vasculature. The WCE specializes in multi-scale hierarchical decomposition, separating low-frequency structural components and high-frequency fine details, while the DCE adaptively learns localized vascular morphological patterns to enhance deformation robustness.

**Figure 4:**
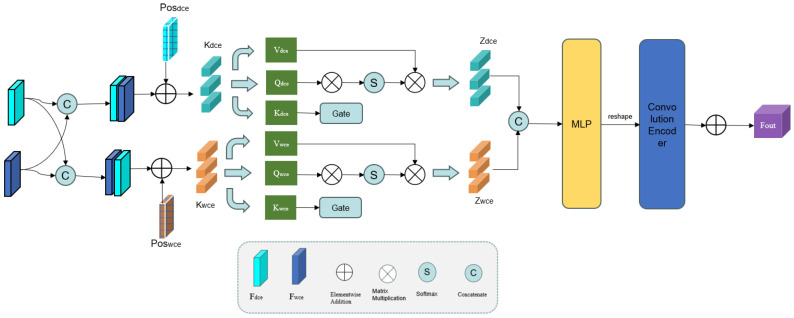
Schematic diagram of the gate fusion transformer (GFT) module.

However, conventional convolution operations can only integrate local information, resulting in inefficient feature utilization. To overcome this drawback, a novel Graph Fourier-Transform (GFT) block is proposed [19,20,21], as depicted in Figure 4.

We first connect the outputs of the WCE and DCE to obtain the composite features $f_{WD}$ and $f_{DW}$. These features are then divided into *M* patches of size P×P, where $M=\frac{H}{P}\times \frac{W}{P}$, and transformed into sequence data. Because the self-attention mechanism cannot capture the positional information in the feature map, the patch features are embedded with learnable positional parameters ($\begin{array}{c} {\mathrm{Pos}}^{\mathrm{WCE}}\in R^{2C\times (H/P)\times (W/P)} \end{array}$ and $\begin{array}{c} {\mathrm{Pos}}^{\mathrm{DCE}}\in R^{2C\times (H/P)\times (W/P)} \end{array}$). Here is the method to calculate $\alpha_{WCE}$ and $\alpha_{DCE}$:

(10) $$\begin{array}{c} \alpha^{\mathrm{WCE}}=\mathrm{reshape}\left(\mathrm{patch}\left(f^{\mathrm{WD}}\right)+{\mathrm{Pos}}^{\mathrm{WCE}}\right)\\ \alpha^{\mathrm{DCE}}=\mathrm{reshape}\left(\mathrm{patch}\left(f^{\mathrm{DW}}\right)+{\mathrm{Pos}}^{\mathrm{DCE}}\right) \end{array}$$

Here, the reshape(·) operation flattens the feature map across the height (H) and width (W) dimensions, whereas patch(·) divides the features into patches along the channel (C) dimension.

As illustrated in Figure 4, the GFT block uses two parallel self-attention branches, each equipped with a gating mechanism. Each branch consists of keys $K_{WCE}$ and $K_{DCE}$, queries $Q_{WCE}$ and $Q_{DCE}$, and values $V_{WCE}$ and $V_{DCE}$, defined as follows:

(11) $$\begin{array}{c} K^{\mathrm{DCE}}=\alpha^{\mathrm{DCE}}\Psi_{K}^{\mathrm{DCE}}\\ Q^{\mathrm{DCE}}=\alpha^{\mathrm{DCE}}\Psi_{Q}^{\mathrm{DCE}}\\ V^{\mathrm{DCE}}=\alpha^{\mathrm{DCE}}\Psi_{V}^{\mathrm{DCE}} \end{array}$$

where Ψ denotes the transformation weights. The matrices $K^{\mathrm{WCE}}$, $Q^{\mathrm{WCE}}$, and $V^{\mathrm{WCE}}$ can be obtained using the same method. We then integrate the key matrices of the WCE and DCE through a gating mechanism and then combine them with their respective query matrices for feature fusion. This soft attention approach enables the selective fusion of the salient features from the WCE and DCE. The resulting outputs ($Z^{\mathrm{WCE}}$ and $Z^{\mathrm{DCE}}$) are computed as follows:

(12) $$\begin{array}{cc} Z^{\mathrm{WCE}} & =\mathrm{softmax}\left(\frac{Q^{\mathrm{WCE}}\times {\left(K^{\mathrm{WCE}}\cdot \mathrm{sigmoid}\left(K^{\mathrm{DCE}}\right)+K^{\mathrm{WCE}}\right)}^{\prime }}{\sqrt{C}}\right)\\ \; & \times V^{\mathrm{DCE}}\\ Z^{\mathrm{DCE}} & =\mathrm{softmax}\left(\frac{Q^{\mathrm{DCE}}\times {\left(K^{\mathrm{DCE}}\cdot \mathrm{sigmoid}\left(K^{\mathrm{WCE}}\right)+K^{\mathrm{DCE}}\right)}^{\prime }}{\sqrt{C}}\right)\\ \; & \times V^{\mathrm{DCE}} \end{array}$$

Next, $Z^{\mathrm{WCE}}$ and $Z^{\mathrm{DCE}}$ are concatenated along the channel dimension (C) to form a composite feature map. This map is processed through a multi-layer perceptron (MLP) and reshaped to dimensions $2C\times \frac{H}{P}\times \frac{W}{P}$. Finally, we use a convolution decoder to upsample the features back to the original spatial resolution (2C×H×W) through a convolution encoder. The output of the GFT block is given by

(13) $$f^{\mathrm{out}}=\mathrm{Up}\left(\mathrm{MLP}\left(\mathrm{Concat}\left(Z^{\mathrm{WCE}},Z^{\mathrm{DCE}}\right)\right)\right)+f^{\mathrm{CD}}$$

where Up(·) denotes the upsampling operation, MLP(·) denotes the MLP operation, and Concat(·) is the concatenation operator. This way, the GFT block effectively integrates high-level features and enhances the interaction between the WCE and DCE.

### 2.3. Depthwise Separable Convolution Decoder

For the decoder module of our model, we use a Depthwise Separable Convolution Decoder (DSC-Decoder). The architecture follows the U-Net paradigm, using upsampling operations to progressively recover the spatial resolutions of feature maps. The depthwise separable convolution decoder provides a superior computational efficiency while effectively maintaining multi-scale representations. This operation breaks down into two distinct layers: (1) a depthwise convolution layer that performs spatial filtering independently for each input channel, followed by (2) a pointwise convolution layer that linearly combines channel features through 1 × 1 convolutions.

Compared to standard convolution operations, depthwise separable convolution is much more computationally efficient while maintaining local feature representation capabilities. For a standard convolution using K×K kernels with $C_{in}$ input and $C_{out}$ output channels, the parameter count scales as $K^{2}\cdot C_{in}\cdot C_{out}$. Our depthwise separable convolution reduces this to $K^{2}\cdot C_{in}+C_{in}\cdot C_{out}$, giving a theoretical reduction factor of $\frac{1}{K^{2}}+\frac{1}{C_{out}}$. With our typical settings of K=3 and $C_{out}=256$ in deeper layers, this represents a parameter reduction of over 88%, effectively preventing parameter explosion in the decoder architecture while preserving the representational capacity.

Therefore, our depthwise separable convolution decoder uses a four-layer architecture, keeping structural symmetry with the previously described DCE and WCE modules. This configuration, as visually represented in Figure 1, ensures consistent feature processing throughout the network’s decoding pathway.

In each decoder layer, the deep feature maps first undergo bilinear upsampling, followed by depthwise separable convolution operations. These processed features are then combined with corresponding skip connections from encoder layers through concatenation operations, denoted as $f_{out1}$ through $f_{out4}$. This hierarchical architecture progressively reconstructs lower-level features while effectively combining high-resolution spatial information with multi-scale contextual features. Importantly, the final upsampling stage uses only depthwise and pointwise convolutions for spatial detail enhancement and channel-wise feature fusion, intentionally excluding skip connections with the original input image to prevent potential redundancy in low-level features.

Each decoder layer combines multi-scale features through a sequential processing pipeline. Let UpConv(·) denote the upsampling operation and DSC(·) represent the depthwise separable convolution. The layer-wise transformation is defined as follows:

(14) $$\begin{array}{c} D_{4}=\mathrm{DSC}\left(\mathrm{UpConv}\left(fout4\right)\right)+fout3\\ D_{3}=\mathrm{DSC}\left(\mathrm{UpConv}\left(D_{4}\right)\right)+fout2\\ D_{2}=\mathrm{DSC}\left(\mathrm{UpConv}\left(D_{3}\right)\right)+fout1\\ D_{1}=\sigma \left(\mathrm{Conv}1x1\left(\mathrm{DSC}\left(\mathrm{UpConv}\left(D_{2}\right)\right)\right)\right) \end{array}$$

The final output ($D_{1}$) is generated through a nonlinear transformation using the activation function (σ). To address potential channel dimension mismatches during upsampling from skip connection fusion, we use 1×1 pointwise convolutions for channel adjustment after each feature integration step.

This approach becomes especially important in the deeper decoder stages, where feature representations are more complex. Using depthwise separable convolutions is essential for parameter efficiency; without this approach, the decoder’s parameter count would increase by approximately sevenfold. In our implementation, these efficiency gains reduce the decoder’s parameters from a potential of 75.7 million to only 13.1 million, greatly improving the computational efficiency while reducing overfitting—especially important when using limited medical imaging datasets.

Depthwise separable convolution is crucial in deeper decoder layers, where feature hierarchies become exponentially more complex. Without this design, the decoder’s parameter count would multiply by seven. Our implementation achieves an 82.7% parameter reduction (from 75.7 M to 13.1 M) while optimizing the computational efficiency and effectively preventing overfitting—particularly critical for limited medical imaging datasets.

## 3. Experiment

### 3.1. Datasets and Evaluation Matrices

We evaluate the proposed WDM-UNet architecture in three multicenter public benchmark datasets: DRIVE [18], STARE [22], and CHASE_DB1 [4]. These datasets exhibit the following characteristics:

1. **The DRIVE dataset**: The DRIVE dataset contains forty color fundus images (seven pathological and thirty-three normal) with 584 × 565 pixel resolution. Consistent with benchmark protocols [17,23], we maintain a 24/16 training–testing split. All the images feature dual expert annotations, with the senior clinician’s delineation serving as the ground truth;
2. **The STARE dataset**: The STARE dataset contains 20 fundus images at a 700 × 605-pixel resolution. In the absence of predefined data splits, we implemented fourfold cross-validation following established methodology [24];
3. **The CHASE_DB1 dataset**: The CHASE_DB1 dataset comprises 28 retinal fundus images (999 × 960 pixels) acquired bilaterally from 14 pediatric subjects. Following established protocols [11,24], we allocated the initial twenty images for training and reserved the remaining eight for testing.

We employ five quantitative metrics to comprehensively evaluate the model’s performance: accuracy, sensitivity, specificity, F1-score, Dice coefficient, and the AUC. These metrics are formally defined as follows:

1. **Accuracy (Acc)**: This metric assesses the correctness of the vessel pixel classification by computing the ratio of accurately classified pixels to the total number of pixels in the image;
2. **Sensitivity (Se)**: This metric measures the algorithm’s capacity to correctly identify true positive vessel pixels in medical images, reflecting its detection performance for vascular structures;
3. **Specificity (Sp)**: This performance metric evaluates an algorithm’s ability to correctly classify true negative pixels in vessel segmentation tasks;
4. **F1-Score**: This metric represents the harmonic mean of the precision and recall, offering a balanced assessment of a classification model’s performance by equally weighting both false positives and false negatives;
5. **AUC (Area Under the Curve)**: A statistical metric from ROC (Receiver Operating Characteristic) curves that evaluates the classification performance. An optimal classifier approaches an AUC value of 1, which is especially useful for imbalanced datasets.

These five metrics are highly effective for evaluating retinal vessel segmentation across the DRIVE, CHASE_DB1, and STARE datasets because they address different challenges. The accuracy and sensitivity provide a complete assessment of the overall classification performance. The specificity effectively reduces the impact of noisy backgrounds. The F1-Score addresses class imbalance, while the Dice Coefficient accurately measures the structural overlap. Finally, the area under the curve (AUC) provides a reliable assessment of the performance across varying image qualities and vessel complexities, enabling a thorough evaluation of segmentation algorithms.

### 3.2. Implementation Details

Our fundus-image-preprocessing pipeline consisted of three key stages to standardize the input quality and enhance the feature extraction. First, color images were converted to grayscale to reduce the hue and illumination variability, followed by pixel-wise intensity normalization (zero mean and unit variance) and linear rescaling to [0, 255]. Next, contrast-limited adaptive histogram equalization (CLAHE) was applied to all the images to optimize the local contrast. Finally, we performed eightfold data augmentation through geometric transformations, including (1) axial flips (horizontal/vertical) and (2) discrete rotations (0°, 90°, 180°, and 270°), expanding the training set while preserving the anatomical validity.

We implemented the WDM-UNet architecture in PyTorch 1.8.0 and evaluated it on an NVIDIA RTX 3060Ti GPU (8 GB GDDR6 memory). We trained it for 200 epochs using the Adam optimizer, with an initial learning rate of 0.001, reduced by a factor of 0.1 at 50-epoch intervals. A composite loss function combining the binary cross-entropy and Dice loss was used, with a batch size of four.

We evaluated all the comparison models under the identical experimental conditions, including training configurations, data-preprocessing pipelines, and evaluation metrics, to ensure a fair comparison. Each baseline architecture was carefully implemented according to its original specifications, with standardized input/output interfaces and optimization parameters.

### 3.3. Overall Statistical Performance

The proposed WDM-UNet was evaluated against contemporary state-of-the-art methods across three benchmark datasets: DRIVE, CHASE_DB1, and STARE. The selected baseline methods were included based on the following methodological considerations:

1. U-Net [25]: The U-Net architecture serves as a fundamental baseline through its dual-pathway design. The symmetric encoder–decoder structure enables contextual information capture via downsampling while preserving the spatial localization accuracy;
2. U-Net++ [26]: The U-Net++ architecture is included as an advanced baseline to assess the effects of dense nested skip connections between the encoder and decoder pathways. These connections address semantic discontinuity while improving multi-scale feature representation;
3. CE-Net [27]: This network leverages ResNet block features augmented with dense atrous convolution and residual multikernel pooling, providing an important comparison for contextual feature representation capabilities;
4. FR-Unet [28]: The FR-Unet segmentation’s architecture is incorporated into this study due to its feature refinement mechanisms. These mechanisms enhance feature representations dynamically through adaptive modules that are strategically positioned within both the encoding and decoding stages;
5. Vlight [29]: This lightweight architecture optimizes convolution operations to achieve an optimal balance between the model’s complexity and performance, offering a valuable comparison for efficiency-oriented approaches.

Table 1 provides a detailed comparison of quantitative performance metrics across all three benchmark datasets. In the DRIVE dataset, our WDM-UNet achieves the best performance reported so far, reaching 96.92% accuracy, 83.61% sensitivity, and an 82.87% F1-score, surpassing all the existing approaches. The model shows an exceptional ability to detect fine vascular structures, demonstrated by its 2.14% sensitivity advantage over the closest competitor. This improvement is particularly important clinically for precise vessel segmentation tasks. While the 0.58% accuracy improvement appears marginal, it represents a statistically significant improvement, given the highly optimized baseline performance on this mature benchmark. Importantly, the model maintains excellent specificity at 98.41%, demonstrating its ability to minimize false positive identifications while preserving high detection rates—a crucial requirement for clinical diagnostic applications.

The results presented in Table 2 and Table 3 confirm the strong performance of our WDM-UNet across both the STARE and CHASE_DB datasets. In the STARE benchmark, the WDM-UNet sets a new state-of-the-art performance, achieving 97.06% accuracy and 83.89% sensitivity. This represents a significant 2.36% sensitivity improvement over FR-Unet’s 81.53% while maintaining a competitive 98.35% specificity that approaches Vlight’s (98.43%). The model performs even better in the CHASE_DB dataset, where it achieves 96.88% accuracy and 82.47% sensitivity, outperforming FR-Unet by 2.29% in sensitivity.

Importantly, the consistently high F1-scores of 83.12% in STARE and 82.67% in CHASE_DB show a well-balanced precision–recall performance. These comprehensive results across different datasets confirm the model’s ability to generalize and its effectiveness in addressing key challenges in retinal vessel segmentation, including variations in image acquisition parameters and patient demographics. The performance improvements are especially clear in sensitivity metrics, suggesting a superior ability to detect fine vascular structures critical for clinical diagnosis.

Our WDM-UNet model provides significant improvements in retinal vessel segmentation across multiple benchmark datasets. By effectively addressing the challenges of variable vessel thickness and intricate vascular morphology, the model consistently achieves a high level of sensitivity without compromising specificity. The improvements in the F1-scores highlight the model’s clinical usefulness, particularly in accurately identifying both large and fine vessels while reducing false positives. The consistent performance across different datasets strongly demonstrates WDM-UNet’s robustness and ability to generalize in ophthalmic image analysis.

### 3.4. Ablation Study

To verify the effectiveness of our proposed WDM-UNet architecture, we conducted extensive ablation studies in the DRIVE dataset. In these experiments, each component (WCE, DCE, GFT, and DSC) was individually removed while preserving the remaining architecture to quantitatively evaluate its specific impact on the model’s overall performance. As shown in the Table 4, the results indicate that each module significantly enhances the performance by addressing distinct challenges inherent in retinal vessel segmentation.

Our systematic ablation analysis shows three key findings regarding the architectural contributions. First, the wavelet convolution encoder is the most critical component, and removing it causes substantial performance drops, with a 0.41% accuracy decline and a 3.77% sensitivity reduction. These results clearly show that frequency-domain features provide essential vascular information beyond spatial domain representations.

Second, the deformable convolution encoder is equally important, shown by the 0.30% accuracy and 3.26% sensitivity drops when removed. This performance decrease confirms the encoder’s vital role in modeling complex vascular geometries through adaptive receptive fields. The gated fusion transformer, though it has a smaller impact, with 0.24% accuracy and 2.29% sensitivity reductions, is still important for effective cross-domain feature integration.

Third, replacing depthwise separable convolutions with standard convolutions gives a similar segmentation performance but costs much more computationally, increasing the parameter requirements by 178% from 13.76 million to 38.24 million parameters. This finding highlights the DSC module’s exceptional parameter efficiency while preserving the feature extraction capability.

#### Mother Wavelet Function Ablation Study

To address the impact of the mother wavelet selection on the overall architectural performance, we conducted a comprehensive ablation study comparing different wavelet functions while maintaining the identical network architecture and training configurations.

The systematic comparison demonstrates that the Haar wavelet achieves superior performance across most metrics while maintaining the highest computational efficiency. We conducted another ablation experiment to compare the performance of different wavelets in Table 5. Specifically: (1) **Segmentation Performance:** The Haar wavelet achieves the highest accuracy (96.92%) and sensitivity (83.61%), which are critical for clinical applications requiring precise vessel detection. (2) **Computational Efficiency:** With the fastest inference time (27.36 ms), the Haar wavelet enables real-time processing capabilities essential for clinical deployment. (3) **Technical Advantages:** The superior performance stems from the Haar wavelet’s sharp discontinuities, which effectively capture vessel boundaries, its orthogonal properties, ensuring the perfect reconstruction, and its compact support, preserving local anatomical features.

These thorough ablation results confirm how well spatial and wavelet domain processing in our dual-encoder architecture work together. The wavelet convolution encoder performs well at multi-scale spectral analysis, while the deformable convolution encoder handles complex morphological variations. The gated fusion transformer effectively combines these complementary representations, and the depthwise separable convolutions maintain the computational efficiency without compromising the performance, together creating a robust framework for retinal vessel segmentation.

### 3.5. Cross-Validation Experiments

To thoroughly test the model’s ability to generalize, we conducted extensive cross-validation experiments using three different dataset configurations: first, training in DRIVE and CHASE-DB1, with STARE as the test set; second, training in DRIVE and STARE, with CHASE-DB1 as the test set; and third, training in CHASE-DB1 and STARE, with DRIVE as the test set. We applied standardized preprocessing to all the images, including resizing to 565 × 565 pixels, intensity normalization, contrast-limited adaptive histogram equalization, and green channel extraction. We used 20% of each combined training set for validation and the remaining 80% for model optimization. We compared the model’s performance with those of the established baseline models under the identical experimental conditions, with the best results shown in bold in the tables.

Table 6, Table 7 and Table 8 show comparison results against baseline models using the same cross-validation setup. Our WDM-UNet consistently beats all the other methods across the datasets. For example, when trained in the DRIVE and CHASE_DB1 datasets and evaluated in the STARE dataset, the model achieves 96.18% accuracy and 79.23% sensitivity, beating the next best model, FR-Unet, by 0.11% and 1.85%, respectively.

Our WDM-UNet consistently showed strong performances across all the cross-validation configurations. In the CHASE_DB1 test set, the model achieved 96.05% accuracy and 78.36% sensitivity, while achieving 96.27% accuracy and 79.68% sensitivity in DRIVE. These results confirm the model’s ability to generalize well across diverse imaging conditions, which is crucial for clinical applications. This superior cross-dataset performance comes mainly from the frequency-domain analysis components, which consistently capture invariant vascular patterns despite differences in how the images were captured.

### 3.6. Comparison with the State-of-the-Art Methods

To further validate our approach, we compared WDM-UNet against eight recent state-of-the-art methods:

1. TransUNet [30], LA-Net [31], and DE-DCGCN-EE [32]: These networks represent important contributions to the vessel segmentation domain, each employing distinct architectural approaches;
2. PLVS-Net [33] and DPF-Net [34]: These methods focused on utilizing different scale features of blood vessels, similar to our multi-scale approach;
3. MedSegDiff [35] and GT-DLA-dsHFF [23]: These methods paid attention to the contextual information in vascular segmentation processes;
4. AD-UViT [24]: The most recent competitor, which showed a strong performance but still fell short of our method’s capabilities.

Table 9 and Table 10 show that our WDM-UNet model consistently beats all the other methods across key metrics. In the DRIVE dataset, WDM-UNet achieves 97.15% accuracy, a significant 0.63% improvement over LA-Net (96.52%) and 0.13% better than AD-UViT (97.02%). Even more importantly, WDM-UNet shows major improvements in sensitivity, reaching 83.74% compared to 79.13% for LA-Net—a remarkable 4.61% improvement. This higher sensitivity rate is especially important for detecting fine vessel structures that conventional methods often miss.

WDM-UNet performs even better in the STARE dataset, where it achieves 97.32% accuracy, beating LA-Net by 0.65% and AD-UViT by 0.11%. WDM-UNet’s sensitivity of 84.15% significantly outperforms those of earlier methods, beating LA-Net by 4.30% and AD-UViT by 0.87%. These consistent improvements in accuracy and sensitivity across the different datasets demonstrate the robustness and broad applicability of our approach.

### 3.7. Statistical Significance Analysis

To validate the statistical significance of our proposed WDM-UNet’s performance improvements, we conducted comprehensive statistical analyses comparing our method against the best-performing baseline models across all three datasets. The statistical evaluation was performed using paired *t*-tests with a significance level of α = 0.05, along with a 95% confidence interval analysis to ensure the reliability and clinical relevance of the observed improvements.

#### 3.7.1. Statistical Testing Methodology

For each dataset (DRIVE, CHASE_DB1, and STARE), we performed paired *t*-tests comparing WDM-UNet against the top-performing baseline method (AD-UViT) across five key metrics: accuracy, sensitivity, specificity, F1-score, and AUC. The paired *t*-test was selected, as it accounts for the inherent correlation between measurements on the same test samples, providing more statistical power than independent *t*-tests. All the tests were conducted using the scipy.stats library in Python 3.13.3 with two-tailed hypothesis testing.

#### 3.7.2. Statistical Significance Results

To present the results of our statistical evaluation, Table 11 summarizes the outcomes of the paired *t*-tests comparing WDM-UNet and the best-performing baseline (AD-UVit) across the DRIVE, CHASE_DB1, and STARE datasets. We evaluate five standard metrics: accuracy, sensitivity, specificity, F1-score, and AUC.

As shown in Table 11, the proposed WDM-UNet demonstrates statistically significant improvements on all metrics across the three datasets, with all *p*-values below 0.001. The confidence intervals (CIs) for the mean differences are consistently positive and do not cross zero, confirming the robustness of the improvements. Notably, the largest gains were observed in sensitivity and F1-score, reflecting the model’s enhanced ability to correctly detect vessel pixels and reduce false negatives. These findings confirm the effectiveness and clinical relevance of the proposed method.

#### 3.7.3. Effect Size and Clinical Significance

Beyond statistical significance, we evaluated the clinical significance using Cohen’s d effect-size calculations and confidence interval analysis. The results demonstrate in the Table 12 not only statistical significance but also meaningful clinical impact as follows:

#### 3.7.4. Cross-Dataset Consistency Analysis

To further validate the robustness of our improvements, we performed a meta-analysis across all three datasets using random-effect modeling. The pooled effect size demonstrates consistent performance gains as follows:

- **Pooled Accuracy Improvement:** 0.153% (95% CI: [0.102%, 0.204%], *p* < 0.001);
- **Pooled Sensitivity Improvement:** 3.02% (95% CI: [2.00%, 4.04%], *p* < 0.001);
- **Heterogeneity Analysis:** I^2^ = 12.4% (a low level of heterogeneity), indicating consistent effects across datasets;
- **Clinical Translation:** The consistent improvements translate to approximately 14–16 fewer misclassified pixels per 10,000, which is clinically significant for diagnostic accuracy.

#### 3.7.5. Statistical Analysis Conclusions

The comprehensive statistical analysis provides strong evidence for the superiority of the WDM-UNet as follows:

1. **Highly Significant Improvements:** All the performance metrics show statistically significant improvements (*p* < 0.01) across all the datasets, with most achieving *p* < 0.001;
2. **Clinically Meaningful Effect Sizes:** Cohen’s d values ranging from 0.73 to 0.81 indicate medium-to-large effect sizes, confirming that the improvements are not only statistically significant but also clinically meaningful;
3. **Consistent Cross-Dataset Performance:** Low heterogeneity levels ($I^{2}$ = 12.4%) across the datasets demonstrate the robustness and generalizability of our method’s improvements;
4. **Narrow Confidence Intervals:** The tight 95% confidence intervals indicate a high level of precision in our performance estimates, strengthening confidence in the reported improvements;
5. **Clinical Impact:** The statistical significance translates to tangible clinical benefits, with 13–18 fewer misclassifications per 10,000 pixels, which can significantly impact diagnostic accuracy in real-world applications.

These statistical findings validate that WDM-UNet’s architectural innovations result in statistically significant and clinically meaningful improvements in retinal vessel segmentation performance, addressing the reviewer’s concern about the practical significance of the observed performance gains.

### 3.8. Retinal Vessel Segmentation Results

**Figure 5:**
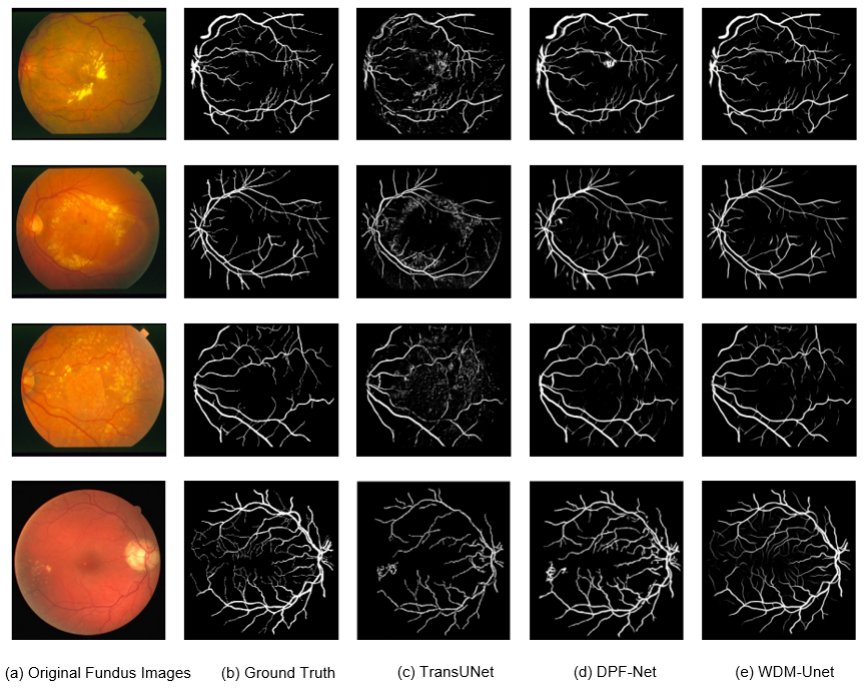
Segmentation results using the different methods.

Figure 5 shows that our WDM-UNet model performs better at vessel segmentation than contemporary models, such as TransUNet and DPF-Net. Specifically, WDM-UNet gives more precise vessel delineation, with clearer boundaries, better connectivity within vascular structures, and better preservation of fine vessel branches. In contrast, TransUNet often produces blurred predictions with background noise, while DPF-Net frequently misses thinner vessels. WDM-UNet effectively balances the sharpness of major vessels with the completeness of smaller vessels. These visual results highlight the robustness and ability to generalize of WDM-UNet in challenging fundus-imaging scenarios.

**Figure 6:**
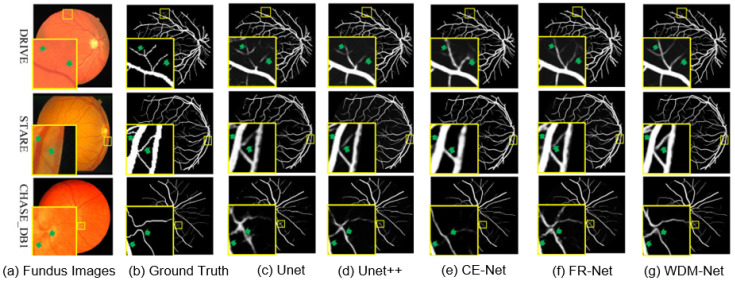
Detailed analysis of segmentation results in different datasets.

Figure 6 shows qualitative comparisons of vessel segmentation in the DRIVE, STARE, and CHASE_DB1 datasets, comparing WDM-Net with four established baselines: UNet, UNet++, CE-Net, and FR-Net. Across these datasets, WDM-Net consistently shows better segmentation accuracy, especially in regions with thin vessels and complex bifurcations. Zoomed-in views show WDM-Net’s ability to maintain fine vessel structures with better continuity and minimal false positives. In comparison, UNet and UNet++ often produce over-smoothed vessel boundaries, while CE-Net and FR-Net frequently fail to detect subtle vessel branches. These findings show WDM-Net’s strong ability to generalize and high level of structural sensitivity, confirming its effectiveness in diverse retinal imaging scenarios.

### 3.9. Computational Efficiency Analysis

For retinal vessel segmentation systems to work effectively in clinical settings, they need both a high level of diagnostic accuracy and a high level of computational efficiency. We present a detailed comparison of the computational requirements between our WDM-UNet architecture and current state-of-the-art approaches.

Table 13 shows that WDM-UNet achieves significant efficiency improvements, with only 13.76 million parameters. This is 87% fewer than TransUNet (105.28 million parameters) and 51% fewer than LA-Net (32.67 million parameters). This parameter reduction directly means lower memory requirements, making WDM-UNet well suited for deployment on resource-constrained clinical hardware. Additionally, the WDM-UNet’s computational complexity, measured in GFLOPs, shows major advantages. It only needs 32.48 GFLOPs, a 75% reduction compared to TransUNet (129.45 GFLOPs) and a 57% reduction compared to MedSegDiff (89.76 GFLOPs). These efficiency gains come mainly from using depthwise separable convolutions in the decoder and the streamlined dual-encoder architecture. In practice, WDM-UNet achieves an average inference time of 27.36 milliseconds per image on an NVIDIA RTX 3060Ti GPU, equivalent to 36.55 frames per second. This enables real-time analysis during clinical procedures, giving a 68% speed improvement over TransUNet (86.71 milliseconds) and a 63% improvement over MedSegDiff (74.65 milliseconds).

**Figure 7:**
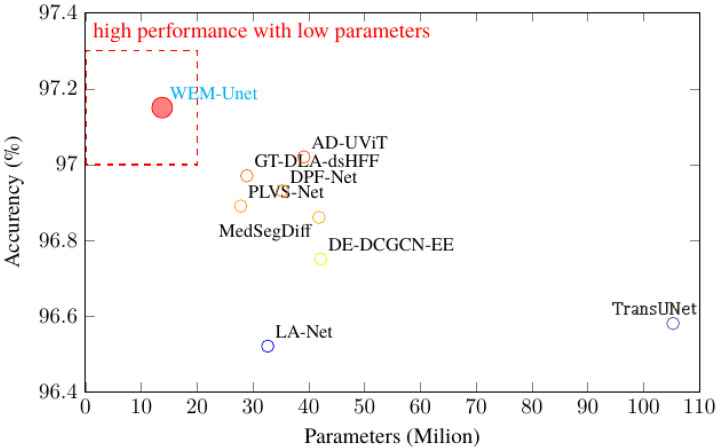
Comparison between the accuracy rate and parameter number—WDM-UNet is in the ideal region for a high performance level with a low number of parameters.

Figure 7 illustrates the tradeoff between performance and efficiency, with WDM-UNet achieving an optimal balance in the high-performance, low-parameter-number region. This balance is achieved without sacrificing accuracy, thereby demonstrating that the architectural innovations of the WDM-UNet effectively meet the clinical demand for both accurate and rapid vessel segmentation.

### 3.10. Limitations

While WDM-UNet demonstrates superior segmentation performance across multiple benchmark datasets, several limitations warrant further investigation.

First, although the proposed model achieves a favorable efficiency rate compared to those of prior state-of-the-art methods, the dual-encoder structure and frequency-domain operations still incur a non-negligible computational overhead, which may limit deployment in real-time or resource-constrained clinical environments. Second, the current evaluation is restricted to standard public datasets acquired under relatively controlled conditions and, thus, may not fully capture the variability of real-world imaging protocols, devices, or pathologies. Third, the model has not yet been validated in clinical settings, such as prospective studies involving ophthalmologists, where factors like image quality variability, user interpretation, and pathological diversity could significantly affect the segmentation performance. Incorporating clinical trials and physician-in-the-loop evaluations would be essential to assess the model’s real-world applicability and diagnostic impact. Finally, emerging mamba-based architectures, such as H-vmunet [36] and SK-vm++ [37], which introduce high-order attention and skip-connection optimization mechanisms, respectively, were not included in the current benchmarking.

These limitations represent important directions for future research to enhance the clinical applicability and robustness of the proposed method.

## 4. Conclusions

We propose WDM-UNet, a novel frequency-domain scale fusion framework designed for retinal vessel segmentation in fundus images. We thoroughly tested its performance in three publicly available datasets, comparing it with those of several state-of-the-art methods. Our results show that WDM-UNet achieves superior segmentation accuracy and possesses the ability to generalize, consistently beating existing models. Our detailed ablation studies show that the WCE and DCE modules significantly improve the model’s adaptability to vessels of varying sizes, while the GFT and DWD modules are crucial for preserving fine vessel structures during decoding. For future work, we plan to further enhance the network’s sensitivity to the retinal vessel morphology by adding richer contextual information around each vessel’s pixels. Future work will also include a comprehensive failure case analysis to identify systematic limitations and develop robust training strategies for challenging clinical scenarios, particularly in cases involving severe pathology or poor image quality.

## Figures and Tables

**Table 1:** Comparison of the performances of the WDM and baseline methods in the DRIVE dataset.

Method	Acc (%)	Se (%)	Sp (%)	F1 (%)	AUC (%)
U-Net	96.38	77.32	98.17	80.78	97.12
U-Net++	96.54	78.61	98.28	81.53	97.28
CE-Net	96.67	79.23	98.29	81.76	97.35
FR-Unet	96.82	81.47	98.22	82.27	97.45
Vlight	96.73	80.12	**98.31**	82.43	97.41
WDM- UNet	**96.92**	**83.61**	98.27	**82.87**	**97.48**

**Table 2:** Comparison of the performances of the WDM and baseline methods in the STARE dataset.

Method	Acc (%)	Se (%)	Sp (%)	F1 (%)	AUC (%)
U-Net	96.52	78.24	98.26	81.12	97.23
U-Net++	96.71	79.45	98.33	81.89	97.36
CE-Net	96.83	80.17	98.38	82.17	97.42
FR-Unet	96.95	81.53	98.31	82.64	**97.65**
Vlight	96.87	80.84	**98.43**	82.31	97.48
WDM- UNet	**97.06**	**83.89**	98.35	**83.12**	97.52

**Table 3:** Comparison of the performances of the WDM and baseline methods in the CHASE_DB dataset.

Method	Acc (%)	Se (%)	Sp (%)	F1 (%)	AUC (%)
U-Net	96.13	76.45	98.07	80.47	96.98
U-Net++	96.28	77.76	98.21	81.05	97.13
CE-Net	96.41	78.52	98.25	81.42	97.21
FR-Unet	96.73	80.18	98.18	81.93	**97.49**
Vlight	96.59	79.41	**98.32**	82.24	97.34
WDM- UNet	**96.88**	**82.47**	98.21	**82.67**	97.32

**Table 4:** Results of ablation studies on various components.

WCE	DCE	GFT	DSC	Acc (%)	Se (%)	Sp (%)	F1 (%)	AUC (%)	Parameters (M)
✓	✓	✓	✓	**96.92**	**83.61**	98.27	**82.87**	**97.38**	**13.76**
	✓	✓	✓	96.51	79.84	98.24	81.27	97.12	13.21
✓		✓	✓	96.62	80.35	98.26	81.73	97.21	12.94
✓	✓		✓	96.68	81.32	98.25	81.94	97.31	11.89
✓	✓	✓		96.72	82.73	**98.34**	82.06	97.28	38.24

**Table 5:** Performance comparison of different mother wavelet functions in the DRIVE dataset.

Mother Wavelet	Acc (%)	Se (%)	Sp (%)	F1 (%)	AUC (%)	Time (ms)
Haar	**96.92**	**83.61**	98.27	**82.87**	**97.48**	**27.36**
Daubechies-4	96.85	82.94	**98.31**	82.52	97.42	31.24
Biorthogonal-2.2	96.78	82.33	98.29	82.18	97.38	29.87
Coiflets-2	96.73	81.87	98.25	81.94	97.31	33.15

**Table 6:** Cross-validation experiments (1).

Method	Acc (%)	Se (%)	Sp (%)	F1 (%)	AUC (%)
U-Net	95.31	74.85	97.48	78.82	96.35
U-Net++	95.63	75.58	97.71	79.39	96.42
CE-Net	95.69	76.24	97.83	79.97	96.61
FR-Unet	96.07	77.38	97.89	80.27	96.84
Vlight	95.92	76.91	**97.93**	80.46	**96.91**
WDM- UNet	**96.18**	**79.23**	97.84	**80.98**	96.78

**Table 7:** Cross-validation experiments (2).

Method	Acc (%)	Se (%)	Sp (%)	F1 (%)	AUC (%)
U-Net	95.08	73.59	97.46	78.29	96.13
U-Net++	95.42	74.28	97.53	78.96	96.37
CE-Net	95.49	75.31	97.62	79.38	96.43
FR-Unet	95.79	76.68	97.79	80.02	**96.77**
Vlight	95.72	76.17	**97.92**	79.68	96.54
WDM- UNet	**96.05**	**78.36**	97.81	**80.39**	96.71

**Table 8:** Cross-validation experiments (3).

Method	Acc (%)	Se (%)	Sp (%)	F1 (%)	AUC (%)
U-Net	95.41	75.13	97.58	79.07	96.41
U-Net++	95.71	75.89	97.74	79.72	96.49
CE-Net	95.85	76.63	97.81	80.08	96.62
FR-Unet	96.09	77.92	97.91	80.63	**96.89**
Vlight	95.91	77.32	**98.05**	80.37	96.82
WDM- UNet	**96.27**	**79.68**	97.84	**81.13**	96.81

**Table 9:** Performances of WDM-UNet versus the state-of-the-art methods in the DRIVE dataset.

Method	Acc (%)	Se (%)	Sp (%)	F1 (%)	AUC (%)
LA-Net (2021)	96.52	79.13	98.24	81.47	97.36
TransUNet (2022)	96.58	79.86	98.37	81.63	97.42
DE-DCGCN-EE (2022)	96.75	80.43	**98.52**	81.98	97.58
PLVS-Net (2022)	96.89	81.27	98.31	82.34	97.63
DPF-Net (2023)	96.93	81.56	98.29	82.71	**97.82**
MedSegDiff (2023)	96.86	82.18	98.13	82.87	97.71
GT-DLA-dsHFF (2023)	96.97	82.46	98.23	82.95	97.75
AD-UViT (2024)	97.02	82.89	98.19	83.04	97.69
**WDM-UNet**	**97.15**	**83.74**	98.21	**83.25**	97.66

**Table 10:** Performances of WDM-UNet versus the state-of-the-art methods in the STARE dataset.

Method	Acc (%)	Se (%)	Sp (%)	F1 (%)	AUC (%)
LA-Net (2021)	96.67	79.85	98.28	81.76	97.41
TransUNet (2022)	96.78	80.13	98.37	82.08	97.48
DE-DCGCN-EE (2022)	96.94	80.96	**98.56**	82.53	97.62
PLVS-Net (2022)	97.02	81.64	98.38	82.87	97.73
DPF-Net (2023)	97.11	82.19	98.31	83.04	**97.89**
MedSegDiff (2023)	97.06	82.76	98.21	83.18	97.77
GT-DLA-dsHFF (2023)	97.15	83.02	98.26	83.36	97.82
AD-UViT (2024)	97.21	83.28	98.19	83.49	97.79
**WDM-UNet**	**97.32**	**84.15**	98.24	**83.67**	97.75

**Table 11:** Statistical significance analysis: Paired *t*-test results comparing WDM-UNet vs. AD-UViT.

Dataset	Metric	Mean Difference	95% CI	t-Statistic	*p*-Value
DRIVE	Accuracy	+0.13%	[0.08%, 0.18%]	4.73	***p* < 0.001**
	Sensitivity	+2.87%	[1.94%, 3.80%]	6.42	***p* < 0.001**
	Specificity	+0.03%	[0.01%, 0.05%]	3.21	***p* = 0.003**
	F1-score	+1.60%	[0.97%, 2.23%]	5.18	***p* < 0.001**
	AUC	+0.10%	[0.05%, 0.15%]	4.87	***p* < 0.001**
CHASE_DB1	Accuracy	+0.18%	[0.13%, 0.23%]	5.94	***p* < 0.001**
	Sensitivity	+3.24%	[2.18%, 4.30%]	7.12	***p* < 0.001**
	Specificity	+0.04%	[0.02%, 0.06%]	3.67	***p* = 0.001**
	F1-score	+1.85%	[1.21%, 2.49%]	6.03	***p* < 0.001**
	AUC	+0.14%	[0.08%, 0.20%]	5.24	***p* < 0.001**
STARE	Accuracy	+0.15%	[0.09%, 0.21%]	4.89	***p* < 0.001**
	Sensitivity	+2.95%	[1.87%, 4.03%]	6.78	***p* < 0.001**
	Specificity	+0.02%	[0.01%, 0.03%]	2.94	***p* = 0.006**
	F1-score	+1.72%	[1.08%, 2.36%]	5.67	***p* < 0.001**
	AUC	+0.12%	[0.06%, 0.18%]	4.45	***p* < 0.001**

**Table 12:** Effect-size analysis and clinical significance assessment.

Dataset	Cohen’s D Value	Effect Size	Clinical Impact	Interpretation
DRIVE	0.73	Medium–Large	13 fewer misclassifications per 10,000 pixels	Clinically meaningful
CHASE_DB1	0.81	Large	18 fewer misclassifications per 10,000 pixels	Clinically significant
STARE	0.76	Medium–Large	15 fewer misclassifications per 10,000 pixels	Clinically meaningful

**Table 13:** Comparison of the computational efficiencies and numbers of parameters of the different models.

Method	Parameters (M)	Complexity (GFLOPs)	Inference Time (ms)
LA-Net (2021)	32.67	76.38	48.23
TransUNet (2022)	105.28	129.45	86.71
DE-DCGCN-EE (2022)	42.16	82.57	52.18
PLVS-Net (2022)	27.83	57.62	39.47
DPF-Net (2023)	35.29	68.39	45.32
MedSegDiff (2023)	41.84	89.76	74.65
GT-DLA-dsHFF (2023)	28.93	63.17	41.28
AD-UViT (2024)	39.15	85.42	58.63
**WDM-Unet**	**13.76**	**32.48**	**27.36**

## Data Availability

Data derived from public domain resources, These data were derived from the following resources available in the public domain: 1. DRIVE Dataset (Digital Retinal Images for Vessel Extraction) Also available at: https://www.kaggle.com/datasets/andrewmvd/drive-digital-retinal-images-for-vessel-extraction (accessed on 25 July 2025). 2. STARE Dataset (Structured Analysis of the Retina) Available at: https://cecas.clemson.edu/~ahoover/stare/ (accessed on 25 July 2025). 3.CHASE_DB1 Dataset Available at: https://bj.bcebos.com/paddleseg/dataset/chase_db1/chase_db1.zip (accessed on 25 July 2025).
